# Numerical Model of SPAD-Based Direct Time-of-Flight Flash LIDAR CMOS Image Sensors

**DOI:** 10.3390/s20185203

**Published:** 2020-09-12

**Authors:** Alessandro Tontini, Leonardo Gasparini, Matteo Perenzoni

**Affiliations:** Fondazione Bruno Kessler, Via Sommarive 18, 38123 Trento, Italy; tontini@fbk.eu (A.T.); gasparini@fbk.eu (L.G.)

**Keywords:** Montecarlo, Light Detection and Ranging (LIDAR), Complementary Metal-Oxide Semiconductor (CMOS), Single Photon Avalanche Diode (SPAD)

## Abstract

We present a Montecarlo simulator developed in Matlab^®^ for the analysis of a Single Photon Avalanche Diode (SPAD)-based Complementary Metal-Oxide Semiconductor (CMOS) flash Light Detection and Ranging (LIDAR) system. The simulation environment has been developed to accurately model the components of a flash LIDAR system, such as illumination source, optics, and the architecture of the designated SPAD-based CMOS image sensor. Together with the modeling of the background noise and target topology, all of the fundamental factors that are involved in a typical LIDAR acquisition system have been included in order to predict the achievable system performance and verified with an existing sensor.

## 1. Introduction

Three-dimensional (3D) imaging enables spatial perception for a broad set of application fields, such as personal smart devices, the entertainment industry, industrial production environments, and autonomous vehicles and spacecrafts. Several techniques exist to extract the depth of the objects in the scene [1] and, in this paper, we focus on direct Time-of-Flight (D-ToF) systems.

Time-of-flight (ToF) systems extract the distance estimating the time the light takes to travel from a proper pulsed or modulated emitter to the target and then back to a time-resolved photon detector. With respect to other techniques, they build highly accurate and precise 3D images at high rates and at a relatively low cost while using solid-state image sensors in Complementary Metal-Oxide Semiconductor (CMOS) technology. ToF systems are classified in direct (D-ToF) and indirect (I-ToF) systems. In I-ToF systems, the depth information is estimated by measuring the phase-shift of the received pulsed or modulated light with respect to the emitted one. This is typically achieved by means of photo-demodulators implemented at the pixel level and, in practice, it requires the acquisition of three or more sub-frames, acquired in series and properly combining modulation/demodulation schemes to remove the background, measure the target reflectivity, and then estimate its depth. I-ToF is particularly suitable for short-range, high resolution 3D imaging [2,3,4,5], even in the presence of a strong background (limited by pixel full well capacity). On the other hand, the maximum measurable distance is limited by the modulation period, the precision degrades with distance, and the serial acquisition of the sub-frames introduces artifacts for fast moving objects [6], unless multiple taps are integrated in each pixel [7].

D-ToF exploits the high time resolution provided by avalanche detectors to directly measure the time of flight of the emitted light pulse. With respect to I-ToF techniques no ambiguity is present, thus it can provide longer measurement ranges (only limited by the available optical power budget) and improved robustness against artifacts, since the operating conditions are constant in time. On the other hand, it suffers the presence of strong background light, which can be mitigated by the implementation of smart techniques at the pixel-level for the rejection of uncorrelated light. Recent advances in Single-Photon Avalanche Diode (SPAD) detectors in CMOS technology show that high-sensitivity, low-noise devices can be combined with dense logic in small areas taking advantage of advanced process nodes, down to 40 nm [8], even in combination with 3D stacking [9], and the race towards a fully autonomous car driving system has raised an enormous interest in SPAD-based LIDAR technology.

**Figure 1:**
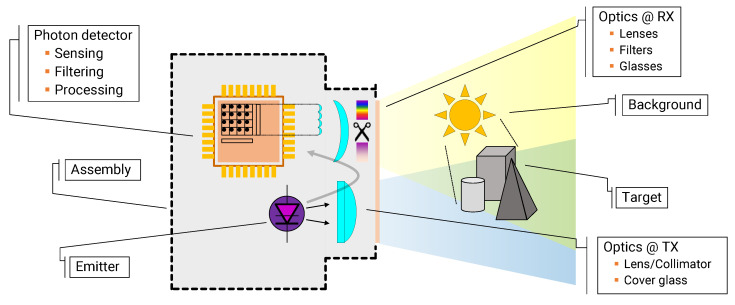
Block diagram of a D-ToF system.

Figure 1 shows an example structure of a SPAD-based D-ToF system, which includes the pulsed light emitter, the time-resolved photon detector, the optical elements to illuminate the target and collect the light echo, the target to be measured, and the environment in which the target is placed.

The core of such a 3D imaging system is represented by a SPAD-based CMOS image sensor, where each pixel is able to extract a distance measurement from the elapsed time between the emission of a laser pulse and the detection of the reflected light. However, each application has its own set of specifications, which leads to the design of very different systems concerning both the architecture of the CMOS sensor and the set of external components such as optical elements and laser source. The performance of such systems are not easily predictable, in particular when the design of the single pixel is not trivial and aims at introducing smart features like background rejection [10,11,12,13,14], interference suppression [15], or partial on-chip memory/histogramming [16,17,18,19]. Additionally, expensive solutions, like 3D stacking [8,9,20] and microlenses [21], are to be carefully evaluated before being employed. Consequently, it is desirable to tailor the design in order to meet the required specifications avoiding the risk to obtain either poor or overkilling performance. For the aforesaid motivations, the availability of a system simulator in order to help the preliminary design phase is of great importance [22,23]. This paper presents the design of a Montecarlo simulator with the aim to emulate the output of a direct time-of-flight (D-ToF) SPAD-based CMOS flash LIDAR sensor. The model is based both on analytical equations modeling the optical concept and the target topology, including the background light and a numerical Montecarlo engine that generates Poissonian events modeling the impinging photons. Starting from the timestamps of the generated photons, many pixel structures can be simulated giving the designers the freedom to foresee the most suited architecture to fullfil the requested specifications. This paper is organized, as follows. Section 2 provides a functional overview of the simulator structure. Subsequently, the most important simulator features are discussed in Section 3 and Section 4. In particular, Section 3 provides the analytical description of the optical system and the discussion of the methodology used to model the emitter power envelope. Section 4 is focused on the Montecarlo simulator with the generation of SPAD-related events and the description of a method to time-compensate timestamps that are generated from the Poisson distribution in order to meet the asynchronous SPAD detection paradigm. Eventually, a procedure to evaluate the performance of the simulated system is described. Section 5 reports the comparison between the proposed simulator and an already existing sensor. Final conclusions are drawn in Section 6.

## 2. Simulator Architecture Overview

**Figure 2:**
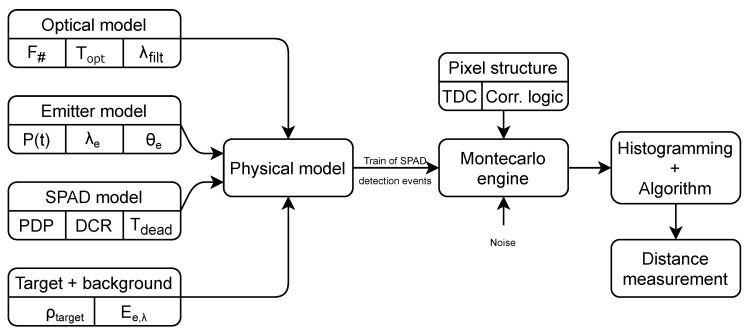
Numerical model architecture and main building blocks.

Figure 2 reports the block diagram representing the simulator structure with its main components. The simulator models the flash LIDAR system by generating random arrival times of detected photons. The first step toward the simulation of a single ToF measurement is the computation of the optical power budget, which mainly depends on the physical properties of the system and the environment: optics (a lens with a given $F_{\#}$, a certain transmittance $\tau_{opt}$ and a bandpass filter with bandwidth $\lambda_{filt}$), emitter (with a given power envelope P(t), a wavelength $\lambda_{e}$ and a beam divergence $\theta_{e}$), target features (assuming Lambertian surface with reflectivity $\rho_{target}$), and spectrum of background light $E_{e,\lambda }$. Through the computation of the optical power budget, it is possible to obtain the optical flux on the array focal plane. Starting from the knowledge of the optical flux, it is possible to generate the time envelope of detected events. In particular, photons can be modeled as a Poisson process, where the number of events in a time *T* follows a Poisson distribution, and the inter-arrival times are exponentially distributed. According to the number of detectors per pixel, a train of SPAD detection events is therefore created, which will be processed by the pixel architecture. The SPAD detector is identified by its fundamental parameters: Photon Detection Probability (PDP), Dark Count Rate (DCR), and, according to the quenching scheme, a certain dead-time $T_{dead}$. The distance information is retrieved by the pixel through a timestamping circuit, usually a Time-to-Digital Converter (TDC) or a Time-to-Amplitude Converter (TAC), that measures the timing information of the impinging photons. However, other features are usually implemented in order to reduce the impact of background light, such as time gating, photon correlation in time domain, and multiple laser shots within the same acquisition window to increase the frame rate. Subsequently, the Montecarlo engine will produce a single ToF value through the elaboration of the incoming train of detected photons by the pixel structure under investigation. Eventually, a histogram of ToF values is created and a distance extraction algorithm is applied in order to produce the final distance measurement.

## 3. System Features Modeling

The aim of this Section is to provide details and mathematical background regarding the modeling of the building blocks of a D-TOF LIDAR system. First, the optical system is modeled to provide analytical formulas for the computation of the impinging optical power on each detector pixel. Subsequently, the modeling of the illumination source is investigated and a methodology is proposed in order to reconstruct the laser emission power envelope.

### 3.1. Optical Model

The amount of optical power hitting the SPAD-based detector is due to the contribution of the illumination source, the background light and the target reflectivity. The optical model is developed considering the computation of the optical power budget for a single pixel, which can be easily extended to the entire array upon the knowledge of the illumination pattern employed. The standard case considers the illumination pattern to be matched along the detector field of view in both horizontal and vertical direction. Different or more sophisticated approaches can be employed and easily taken into account in the optical model through the computation of the optical power density on the target surface. The developed optical model takes the following parameters into account:

- Target topology, assumed to be a Lambertian surface with a given reflectivity value $\rho_{target}$.
- Illumination source, a laser with emitted pulse power $P_{tx}$, divergence $\theta_{e}$, wavelength $\lambda_{e}$ and square or circular spot shape.
- Optical system, composed of a lens with f-number $F_{\#}=f_{lens}/d_{lens}$ and a field of view over the horizontal ($FOV_{H}$) and vertical ($FOV_{V}$) direction.
- Ambient optical power density due to the background light level, $E_{e,\lambda }$.

The power reaching the pixel can be expressed as the amount of radiant exitance $M_{lens}$ collected by the lens multiplied by the area which is observed by the pixel on the target side, $A_{scene}$:

(1) $$\begin{array}{cc} P_{pix} & =M_{lens}\cdot A_{scene} \end{array}$$

The term $M_{lens}$ of Equation (1) is computed considering the target to diffuse a certain radiant exitance $M_{target}$ according to Lambert’s cosine law. Supposing that the target is observed with a lens subtending an angle 2α, the amount of radiant exitance that is collected is obtained from the integration in spherical coordinates:

(2) $$\begin{array}{cc} M_{lens} & =M_{target}\cdot \frac{\int_{0}^{2\pi }\int_{0}^{\alpha }cos\theta sin\theta d\theta d\varphi }{\int_{0}^{2\pi }\int_{0}^{\pi /2}cos\theta sin\theta d\theta d\varphi }\\ \; & =M_{target}\cdot sin^{2}\alpha \end{array}$$

The term $M_{target}$ of Equation (2) is due to the contribution of both the illumination source and the background light. Concerning the illumination source, we consider it to be at a certain distance *z* with a given power $P_{tx}$ and a given divergence $\theta_{e}$ with a square or round spot shape. Concerning the background light, we consider the fraction of the sun irradiance spectrum $E_{e,\lambda }$ through an optical bandpass filter with bandwidth $\left[\lambda_{2}-\lambda_{1}\right]$ that is put on top of the sensor. Because the spectrum of the solar radiation is not constant, the amount of solar radiant flux that can be detected by the sensor is obtained by integration. Consequently, $M_{target}$ equals to:

(3) $$M_{target}=\left\{\begin{array}{cc} \frac{P_{tx}}{4\cdot {\left(z\cdot tan\left(\theta_{e}/2\right)\right)}^{2}} & \mathrm{square}\;\mathrm{spot}\\ \frac{P_{tx}}{\pi \cdot {\left(z\cdot tan\left(\theta_{e}/2\right)\right)}^{2}} & \mathrm{circular}\;\mathrm{spot}\\ \int_{\lambda_{1}}^{\lambda_{2}}E_{e,\lambda }d\lambda =P_{D,BG} & \mathrm{background}\;\mathrm{light} \end{array}\right.$$

**Figure 3:**
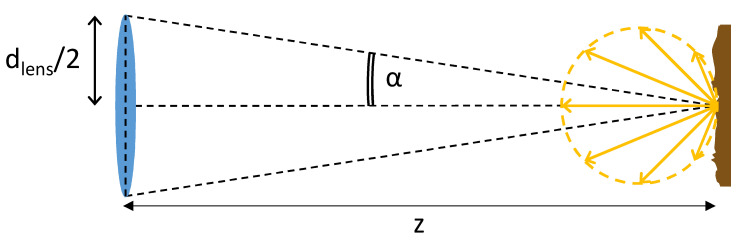
Optical system concept. The light belonging to both the laser emitter and the background is assumed to be diffusely scattered and observed within an aperture of 2α from the collecting lens.

From the analysis of the geometry of Figure 3, the term $sin^{2}\alpha$ of Equation (2) can be expressed as a function of the lens diameter $d_{lens}$ and the target distance *z* as:

(4) $$\begin{array}{cc} sin^{2}\alpha & =\frac{d_{lens}^{2}}{4z^{2}+d_{lens}^{2}} \end{array}$$

The radiant exitance collected by the lens is then given by:

(5) $$\begin{array}{cc} M_{lens} & =M_{target}\cdot \frac{d_{lens}^{2}}{4z^{2}+d_{lens}^{2}} \end{array}$$

The term $A_{scene}$ of Equation (1) can be computed considering the lens to project the area of a M×N array over the target with an aperture angle given by the lens field of view FOV over the horizontal ($FOV_{H}$) and the vertical ($FOV_{V}$) direction:

(6) $$\begin{array}{cc} A_{scene} & =\frac{2z\cdot tan(FOV_{H}/2)}{M}\cdot \frac{2z\cdot tan(FOV_{V}/2)}{N} \end{array}$$

Equation (6) can be rearranged in order to show the pixel area $A_{pix}$ instead of the array dimensions when considering that the lens field of view can be expressed as:

(7) $$FOV=\left\{\begin{array}{cc} 2\cdot arctan(M\cdot Pitch_{H}/2f_{lens})\; & \mathrm{horizontal}\\ 2\cdot arctan(N\cdot Pitch_{V}/2f_{lens})\; & \mathrm{vertical}\; \end{array}\right.$$

The terms $Pitch_{H}$ and $Pitch_{V}$ refers to the physical size of the pixel. By substituting each contribution of the field of view from Equation (7) into Equation (6), we obtain the following expression for the term $A_{scene}$:

(8) $$\begin{array}{cc} A_{scene} & =Pitch_{H}\cdot Pitch_{V}\cdot {\left(\frac{z}{f_{lens}}\right)}^{2}\\ \; & =A_{pix}\cdot {\left(\frac{z}{f_{lens}}\right)}^{2} \end{array}$$

As it can be noticed, the dependence from the array size disappears. Consequently, the computation of the optical budget for each single pixel is valid until the pixel is completely contained within the lens focal plane. While the array size is meant to properly evaluate the impinging power, the simulator works on a single pixel basis: on the single pixel, all of the different conditions of the optical power budget can be set, such as different reflectivity, distance, background, optics parameters, etc. The amount of optical power hitting the pixel is reduced when considering the lens transmittance $\tau_{opt}$ and the target reflectivity $\rho_{target}$, the latter being supposed uniform over the portion of target area which is subtended by the pixel. Alternatively, to model a non-uniform target surface, it is possible to consider an average reflectivity value $\bar{\rho_{target}}$, which is observed by the pixel projection over the target surface. Furthermore, the amount of optical power impinging on the SPAD surface is reduced considering the pixel fill-factor FF. When considering the illumination source with a circular spot shape, we obtain the following expression:

(9) $$\begin{array}{cc} P_{pix,source} & =\frac{\tau_{opt}\cdot \rho_{target}\cdot FF\cdot P_{tx}\cdot A_{pix}}{\pi \cdot F_{\#}^{2}\cdot tan^{2}\left(\theta_{e}/2\right)\cdot \left(4z^{2}+d_{lens}^{2}\right)} \end{array}$$

Considering the background light:

(10) $$\begin{array}{cc} P_{pix,bg} & =\frac{\tau_{opt}\cdot \rho_{target}\cdot FF\cdot P_{D,BG}\cdot A_{pix}\cdot z^{2}}{F_{\#}^{2}\cdot \left(4z^{2}+d_{lens}^{2}\right)} \end{array}$$

Regarding the background light, it is possible to obtain more accurate results by converting the amount of power belonging to each lambda component of the solar radiation spectrum at sea level. With the knowledge of the impinging optical power, it is possible to compute the average photon rate, i.e., the λ parameter of the Poisson process used to statistically model the photon arrival times. The average photon rate is obtained by dividing the power by the photon energy, given by $E_{p}=\frac{h\cdot c}{\lambda_{e}}$, where *h* is the Planck’s constant, *c* is the speed of light (assumed to be in vacuum), and $\lambda_{e}$ is the emitter central wavelength. The average photon rate is translated into an average event rate at the output of the SPAD detector considering two additional parameters: the SPAD photon detection probability (PDP) and the SPAD dead-time. In particular, the SPAD dead-time sets an upper limit to the maximum achievable event detection rate, which may be orders of magnitude lower than the impinging photon detection rate. The effect of SPAD dead-time have been included and extensively discussed in Section 4.

The presence of a scattering medium and its implications on both the two-dimensional (2D) intensity image and the 3D depth map has not been considered in this work. Anyway, regarding the 3D depth map, it can be partly included as an attenuation factor [24] in Equations (9) and (10), and as a spread in time of the received laser echo [25]. However, the potential blurring effect on the 2D image cannot be included as the simulator works considering a single pixel.

### 3.2. Illumination Source-Modeling of the Laser Emission Profile

**Figure 4:**
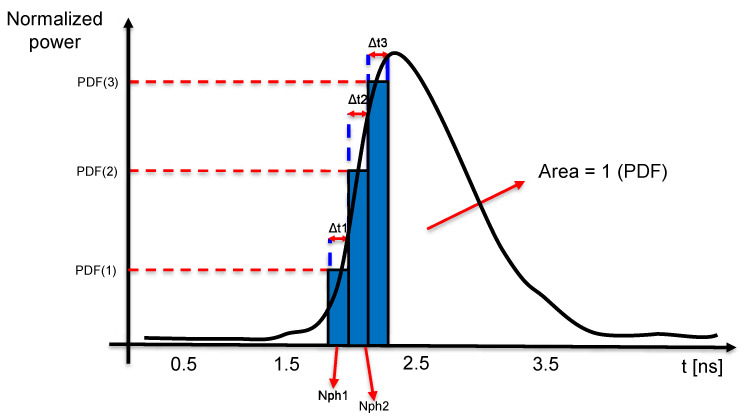
The area of the laser power envelope represents the emitted pulse energy, which is modeled as a probability density function giving the photon flux for each discretized time bin.

At a first approximation, it is possible to consider the illumination source to provide a constant power during the pulse time interval. However, more realistic results can be obtained if the laser illumination source is modeled in order to provide the true time envelope of the photons emission. The underlying inhomogeneous Poisson process has been simulated starting from the laser power emission profile (which can be either provided with the emitter datasheet or measured). Figure 4 shows an example of the temporal evolution of the power of a laser emitter as a function of time.

The area of this curve represents the amount of energy emitted per single laser pulse, *E*, which can be turned into the total number of emitted photons: $N_{ph}=\frac{E\cdot \lambda }{h\cdot c}$. The curve has to be normalized to have unitary area, consequently being considered as a probability density function (PDF). Subsequently, the obtained PDF is discretized into N bins, each of them being sufficiently narrow (in the order of some tens of ps) to have sufficient granularity in the reconstruction process. At each bin, we consider the source to emit photons with a rate:

(11) $$\begin{array}{cc} RATE_{i} & =\frac{PDF(i)}{N_{ph}} \end{array}$$

**Figure 5:**
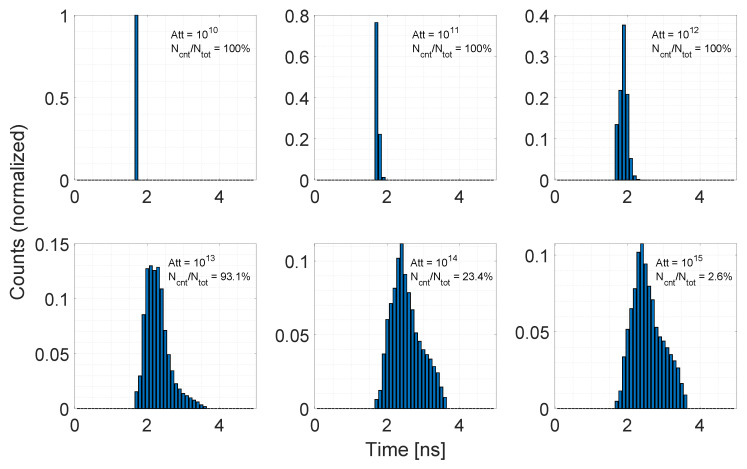
Example of the reconstruction of a laser time envelope. For each sub-figure, the attenuation value and the ratio between detected events and total trials (triggering rate) is reported. Up to $Att=10^{13}$, the reconstructed time envelope is heavily distorted by pile-up effect, where all of the timestamps are compressed into few time bins. For higher attenuation values, the pile-up distortion becomes negligible and it is possible to recover the true time envelope.

For each time bin, we generate one random photon timestamp according to an exponential distribution and this is considered valid if it falls into the corresponding time bin, otherwise it is discarded. By iterating this procedure for a high number of trials, it is possible to reconstruct the laser pulse time envelope. Because we are modeling the photon detection process of a SPAD, it is important to take into account the pile-up effect due to the detector dead time. Being the laser pulse width typically shorter than the SPAD dead time, the true time envelope can be reconstructed if pile-up is avoided, thus being in the single photon regime. Figure 5 shows an example of the experiment run for different power attenuation values. The number of histogram counts have been normalized in order to better show the pile-up effect, where most of the recorded timestamps are compressed toward early values. The attenuation value is calculated as the ratio between the total emitted power and the fraction of power returning back to the pixel (as specified in Section 3.1), and the intensity of pile-up effect is reported for each sub-figure as the average number of detections over total trials. As it can be noticed, the simulator is capable of properly reproducing a variety of detection scenarios, spanning from strong pile-up to photon starved conditions.

The considerations regarding the pile-up effect and the power attenuation values we made in this Section are oriented to the demonstration that the reconstruction method we proposed is able to generate the true laser pulse shape. However, in a real scenario, it is not always possible to recover the true laser pulse envelope. The amount of distortion of the recorded laser pulse echo may introduce systematic errors in the distance detection process, depending on the distance extraction algorithm that is employed. This is of particular importance when distance extraction algorithms that are based on histogram peak detection are used. Referring to Figure 5, a simple peak detection algorithm would provide a detection at t≃1.7 ns with the lowest attenuation value of $10^{10}$, while with the highest attenuation value $10^{15}$ the detection would be located at t≃2.5 ns, resulting in a distance error between the two cases of ≃0.12 m.

## 4. Montecarlo Simulation

In this Section, we first provide a description of the methodology used in order to efficiently generate timestamps from a Poisson process. Subsequently, a discussion on how to time-compensate the generated timestamps in order to meet a precise detection paradigm is provided. Eventually, we discuss important aspects to properly evaluate the results of the simulation process.

### 4.1. Generation of Spad-Related Events

When the intensity of the photon flux is known, it is possible to generate a train of SPAD detection events from the Poisson process. The Poisson approximation is considered valid since the SPAD dead-time $T_{dead}$ is much longer than the coherence time $\tau_{c}$ of the light source, which cannot, therefore, be resolved [26]. The photon interarrival times are generated from the Poisson process (of parameter λ) when considering the probability that the first arrival time *T* is greater than *t*, which is given by:

(12) $$\begin{array}{cc} P(T>t) & =P(N(t)=0)\\ \; & =e^{-\lambda \cdot t} \end{array}$$

The interarrival time *t* is obtained inverting Equation (12):

(13) $$\begin{array}{cc} t & =\frac{-ln(P(T>t))}{\lambda } \end{array}$$

where P(T>t) is replaced with a random number from the uniform distribution in the unit interval U((0,1]). SPAD detection events are then generated from the cumulative sum of *N* interarrival times, up to the desired simulation time. Those events originate from both photon detection mechanisms of illumination source and background light and the generation of dark counts from the SPAD itself. From the simulation perspective, and considering the additive property of Poisson processes, it is possible to generate and superimpose two trains of photon arrival times. The first one contains the background timestamps, from both the environment illumination and dark count events of the detector, with a duration that is equal to the entire measurement window and a parameter $\lambda =\lambda_{bg}+\lambda_{dcr}$. The second one is needed for the photons that belong to the illumination source (with a duration equal to the laser pulse width) with a parameter $\lambda =\lambda_{src}$. However, the photon arrival times generated from the Poisson process needs additional adjustments in order to account for the SPAD dead-time. This effect can be included by removing all events with a time difference smaller than the SPAD dead-time. However, this method is not computationally efficient, since it requires the generation of a certain number of photon arrival times, which will be eventually decimated. Additionally, it is not possible to vectorize the decimation process, thus the efficiency drops due to the need to employ a for-loop. A different method that is computationally efficient avoiding the decimation process and making use of vectorization can be employed. With this method, we generate a maximum number $N_{max}$ of detectable events (i.e., photons detected by the SPAD) within a time interval ΔT when considering the dead time $T_{dead}$:

(14) $$\begin{array}{c} N_{max}=\Delta T/T_{dead} \end{array}$$

Subsequently, a sequence of $N_{max}$ photon arrival times is generated using the result found in Equation (13):

(15) $$\begin{array}{c} t=\left\{t_{0},t_{1},\ldots ,t_{n}\right\},\;n=N_{max}-1 \end{array}$$

Eventually, each photon arrival time is adjusted in order to account for the SPAD dead-time, as follows:

(16) $$\begin{array}{c} t_{i}\to t_{i}+i\cdot T_{dead},\;i=\left\{0,1,\ldots ,N_{max}-1\right\} \end{array}$$

From a mathematical point of view, the proposed method obtains the same result that one would obtain by considering the distribution of interarrival times in the case of a detector deadtime $T_{dead}$:

(17) $$\begin{array}{c} e^{-\lambda \cdot (t-T_{dead})},\;t\geq T_{dead} \end{array}$$

In particular, Equation (17) is valid from the second timestamp on, since (only) the first event can be detected before the detector deadtime $T_{dead}$. Interarrival times are again obtained by inversion:

(18) $$\begin{array}{cc} t & =\frac{-ln\left(P\left(T>t\right)\right)+\lambda \cdot T_{dead}}{\lambda } \end{array}$$

Where, also in this case, P(T>t) is replaced with a random number from the uniform distribution in the unit interval U((0,1]). At this point, the simulator provides the timestamps of the photons detected by the SPAD detector. Usually, a time-to-digital converter (TDC) is used in order to measure the time elapsed between photon detections and provide digital codes that are collected into a histogram. TDCs are mainly characterized by two metrics: quantization error (dependent from the timing resolution) and linearity, in terms of differential nonlinearity (DNL) and integral nonlinearity (INL). The quantization error is included in the model with a rounding operation of the ratio between the photon timestamp and the TDC timing resolution. Regarding the linearity information, it is possible to include their effects upon the knowledge (or a prediction) of the TDC performance that will be used in the final system. In this work, we have included the effect of the differential nonlinearity in order to match the behavior of the TDC used in [13], where a systematic error in the probability of even and odd histogram bins is present. This is included by applying a non-uniform rounding to the generated timestamps, thus artificially increasing the probability of obtaining an even rather than an odd digital code. With the availability of a complete DNL characterization it is also possible to model the behavior of each single TDC code, tailoring the timestamping behavior to the actual implementation. Additional parameters modeling timing uncertainties are also included. In particular, we consider a global jitter value representative of the entire measurement setup, which is drawn from the normal distribution and it is added to the final timing measurement.

### 4.2. Asynchronous Spad Model

**Figure 6:**
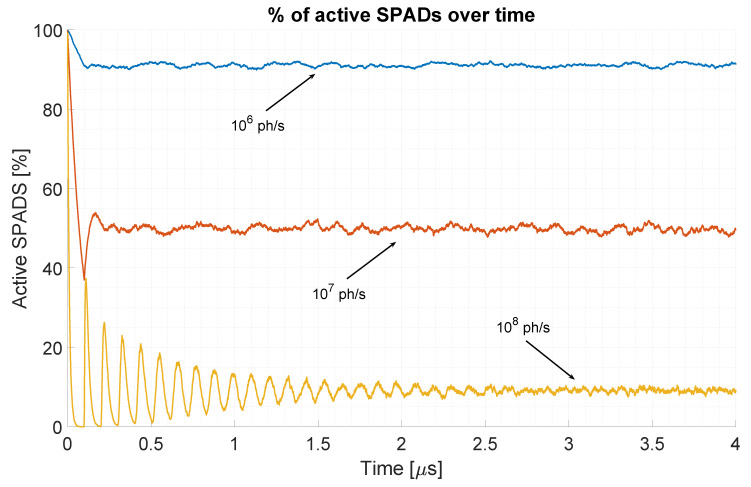
Behavior of a population of SPADs under three different photon flux intensity. At time t=0, all SPADs are active and ready to detect a photon. The average amount of active SPADs is stable after an initial settling time, which depends on the photon flux intensity. The periodicity of the oscillations is given by the SPAD deadtime, which has been set equal to 100 ns.

In this paper, we simulate long-range measurements, in which SPADs are running asynchronously with respect to the observation window, considering either a passive or an active quenching scheme. This allows for a SPAD to detect more than one photon per measurement window and ensures that the average number of SPADs that are active (not in dead time) at any instant is constant over time as opposed to systems in which SPADs are activated synchronously right before the observation window opening. In the latter case, all SPADs are active at the beginning of the measurement; then, some of them will trigger, thus reducing the percentage of active SPADs; after a time equivalent to the SPAD dead time, those SPADs may be recharged (if the sensor allows it) and, thus, be ready to detect another photon. This leads to a percentage of active SPADs that is not constant over time, may oscillate for high photon fluxes, and may temporarily go to zero (all SPADs in dead-time) in extreme cases. In the case of a sensor where SPADs are running asynchronously, the average number of active SPADs (given a certain amount of background flux) is constant over time, except for a transient behavior at the sensor start-up. An example of this behavior is reported in Figure 6, reporting the average number of active SPADs for each time instant when considering that the device is turned on at time t=0. The average number of active SPADs is constant after a certain settling time, which depends on the photon flux intensity, as can be noticed.

**Figure 7:**
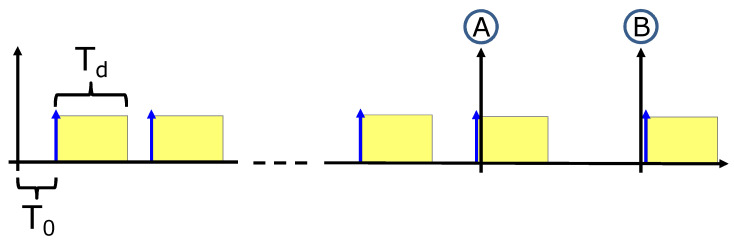
Example of two measurement windows starting at time A and time B. $T_{0}$ is the time of the first detectable event, which is drawn from the Poisson distribution. If the observation starts at time A, $T_{0}$ will be delayed, conversely it will be anticipated if the observation starts at time B.

In order to model a SPAD which is asynchronously operating, i.e., a SPAD that may be at a certain point of its dead time at the beginning of the observation window, we first generate the arrival times of the detected photons (as specified in Section 4.1) and then add an initial offset value generated randomly from a uniform distribution $U(\left[-T_{0},T_{dead}\right])$, where $T_{0}$ is the arrival time of the first generated photon and $T_{dead}$ is the SPAD dead time. This can be intuitively understood referring to Figure 7.

**Figure 8:**
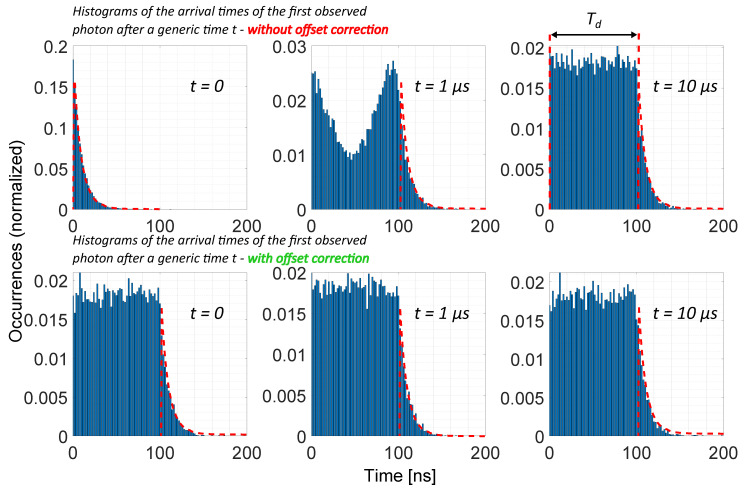
Time-offset compensation to allow the emulation of a asynchronous SPAD detection scheme. The average photon rate has been set to $10^{8}$ ph/s, with a dead time of 100 ns. The histograms are populated with the timestamps of $10^{4}$ events.

By considering time instant “A”, it is possible to notice that the observation starts slightly after a SPAD avalanche. Consequently, the first event will be detected after the SPAD dead time, $T_{dead}$, plus an arrival time ($T_{0}$) that is drawn from the Poisson process. Conversely, referring to time instant “B”, the observation starts slightly before a SPAD avalanche. In this case, the first event will be detected almost instantly, and not after a time $T_{0}$ drawn from the Poisson process. The model has been validated by comparing the distribution of the first timestamp after a generic time *t* in two cases: with and without offset compensation (Figure 8). Because the observation window starts asynchronously and independently from the underlying Poisson process of rate λ, the time distribution is expected to follow a uniform distribution from t=0 up to $t=T_{dead}$, followed by the exponential decrease of the photon’s inter-arrival time ($e^{-\lambda t}$). Without offset compensation, this is only confirmed for values of *t*, which are large with respect to the average photon arrival time. For t=0, we can clearly observe the exponential arrival time distribution, while for values of *t* that are comparable with the average photon arrival time, the distribution is heavily distorted. With offset compensation, the distribution of the first photon timestamp does not depend on time *t*, correctly emulating a detector with asynchronously running SPADs.

### 4.3. Performance Assessment

**Figure 9:**
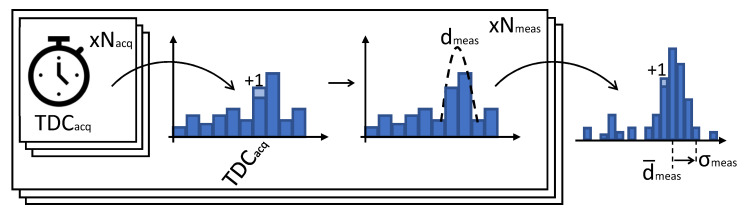
Build process of the histogram of measurements for performance validation.

In the previous Sections, we described the fundamental blocks and the implementation details of the Montecarlo simulator, which results in the availability of a stream of raw timestamps of the detected photons. The strength of the proposed simulator lies in the possibility to test and evaluate the most suitable data processing architecture to extract the final distance measurement. The most common methodology consists in the extraction of one timestamp per acquisition window, which is inserted into a histogram. The distance measurement is then extracted from the histogram after a sufficient number of acquisitions are obtained, in order to increase the signal to noise ratio. In order to increase the data throughput, more complex solutions can be foreseen and tested, such as multiple-event direct histogramming [27,28] and histogram compression techniques [29]. Additionally, it is also possible to evaluate the most suitable distance extraction algorithm, such as peak detection, centroid-based estimation [13], or pulse correlation [30,31]. The final step to assess the performance of the simulated system is to compute a figure of merit that expresses the degree of reliability of each distance measurement. A good candidate is represented by the probability that a given distance measurement can be correct, which can be computed when considering a certain amount of values ($N_{correct}/N_{meas}$). One method that could be employed for this purpose is to use the ground truth to label each measurement as “correct” or “incorrect” and compute the probability straightforwardly. However, in a real case scenario, the knowledge of the ground truth may not be available and alternative methods need to be developed. For this purpose, we define a blind methodology that relies on the self-built statistics obtained from many distance measurements. A single distance measurement is the result of a distance extraction algorithm applied to a histogram populated with many single ToF measurements. Each distance measurement is collected and a histogram of measurements is built giving rise to a quasi-Gaussian distribution of measurements, as depicted intuitively in Figure 9.

**Figure 10:**
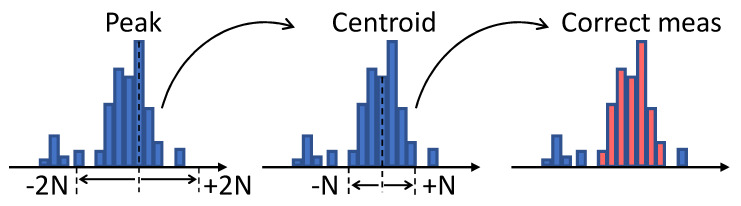
Estimation of the number of correct measurements.

The number of correct measurements $N_{meas}$ is estimated as the sum of ±N bins belonging to the main portion of the bell-shaped distribution. Given a certain bin width, the number *N* is chosen in order to account for a interval of ±3σ, where σ is the expected measurement precision provided as a requirement. First, the position of the distribution centroid is estimated by performing a weighted average over ±2N (thus relaxing the σ requirement) bins around the position showing the highest count. The σ requirement is relaxed only for this first step to account for jitter and other non-idealities in the measurement. This, differently from an average over the whole statistics, ensures excluding the majority of wrong measurements. Subsequently, the estimation of the number of correct measurements is obtained summing ±N bins around the distribution centroid, as depicted in Figure 10.

## 5. Experimental Results

**Figure 11:**
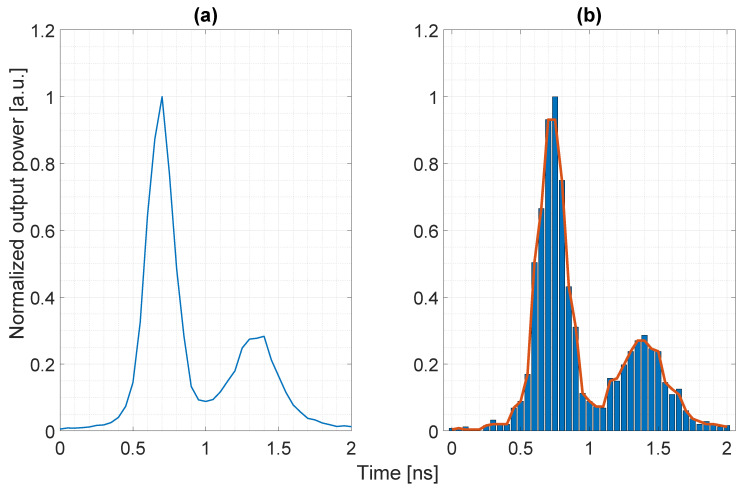
Comparison between (**a**) the measured and (**b**) one realization of the simulated laser pulse envelope (250 ps FWHM, measured energy of ≃6.2 pJ).

The model has been validated against a D-TOF system based on the detector described in [13]. Distance measurements have been carried out indoor in controlled conditions. The simulation parameters, as reported in Table 1, have been tuned in order to match the characteristics of the detector together with the selected optics and laser source. The laser pulse shape has been estimated using a Time-Correlated Single Photon Counting (TCSPC) setup to obtain the temporal envelope of the emitted optical power, as shown in Figure 11. The total emitted energy of the laser pulse has been estimated while using a fast photodiode [32].

**Figure 12:**
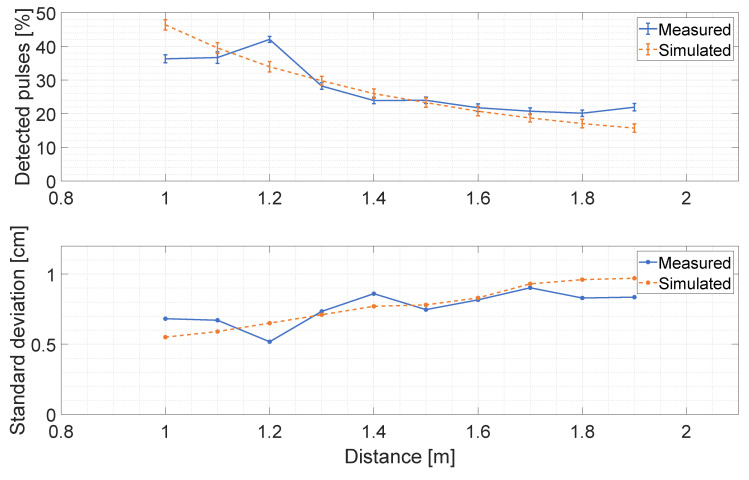
Comparison between measurements and simulations in terms of standard deviation and percentage of detected pulses.

The Montecarlo simulator implements the pixel structure of the detector, which exploits temporal correlation between photon detections in order to improve background rejection. The digital Silicon Photomultiplier (d-SiPM) structure, as described in [13], has been accurately modeled to include the effect of bandwidth limitation due to the OR-tree [13], together with the smart triggering logic that generates the rolling time window in order to detect temporal correlation within the stream of pulses and the differential nonlinearity of the TDC. Due to the very low laser pulse energy available, distance measurements have been carried out within a maximum range of ≃1.9 m using white polytetrafluoroethylene (PTFE) tape as target with ≃75 % Lambertian reflectivity [34]. A comparison that validates the power budget and the Montecarlo photon generation model is provided in Figure 12. The first subplot reports the average number of detected pulses, which is a direct indication of the impinging optical flux. The second subplot reports the measurement precision, computed as the standard deviation of a collection of 250 distance measurements. Each distance measurement, obtained without photon temporal correlation, is extracted from a population (histogram) of 1000 single D-TOF values by applying the algorithm, as described in [13], to both the measured data and simulated data.

**Figure 13:**
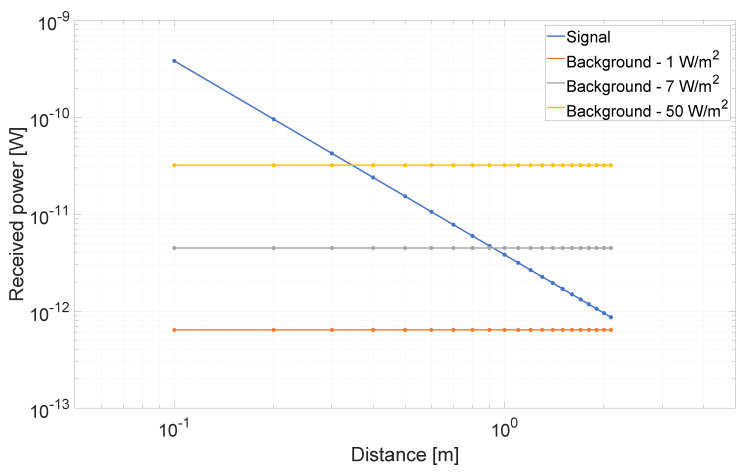
Comparison between the received optical power from signal and four different background illumination intensity.

**Figure 14:**
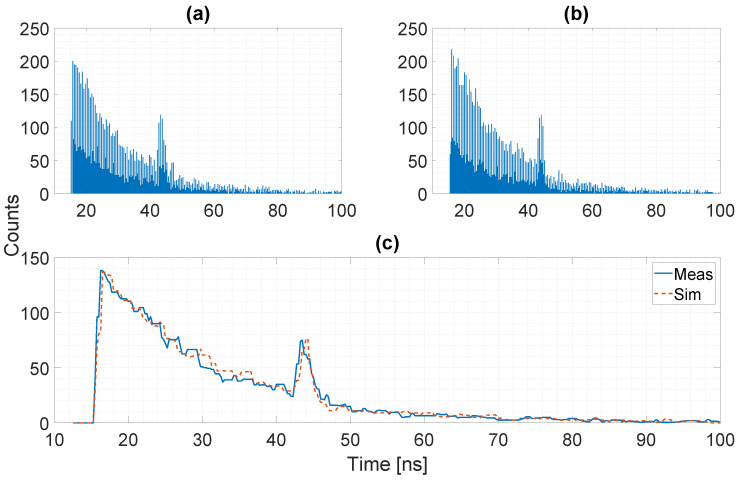
Example of histogram in presence of intense background: measured data (**a**), simulated data (**b**), comparison of the two histograms after median filtering (**c**). The systematic difference between even and odd codes in the histogram originates from the time-to-digital converter (TDC) differential nonlinearity of the detector used for comparison [13], and has been included in the model with good final match.

**Figure 15:**
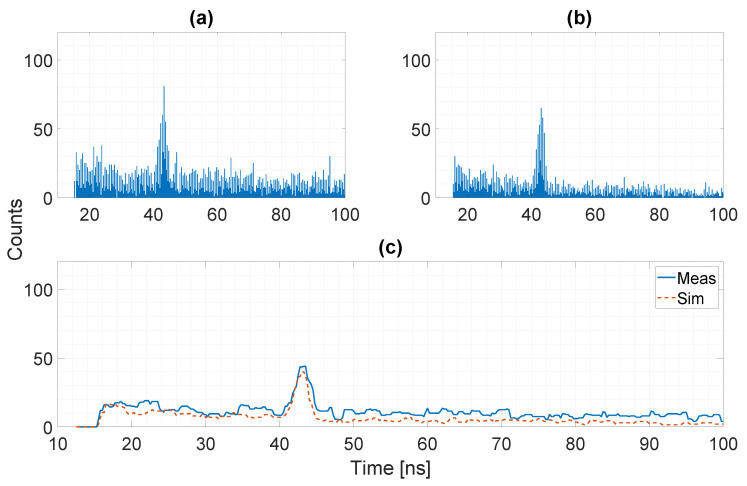
Histograms with temporal correlation of 2 photons within ≃2.3 ns: measured data (**a**) and simulated data (**b**), comparison of the two histograms after median filtering (**c**). The slightly higher noise level in the measured data is due to the presence of additional events due to crosstalk and afterpulsing effects.

A second comparison between measured and simulated data is provided when considering background illumination to exploit the photon correlation logic. In some cases, the signal power level may be orders of magnitude lower with respect to the background power, as depicted in Figure 13, where we show the relationship between signal and background for three different background power density on the target side of 1 W/m^2^, 7 W/m^2^, and 50 W/m^2^. The possibility to exploit photon correlation to drastically reduce the intensity of the background noise is fundamental to recover the signal information. Figure 14 reports one histogram for both measured (a) and simulated (b) data, with a background flux of ≃90 MPh/s/pixel (corresponding to a power density on the target of ≃6.9 W/m^2^) and no correlation between detected photons. Most common algorithms to extract the distance result from the histogram will fail, because, as it can be noticed, the signal peak is below the noise level. The noticeable repetitive noise in the histogram is due to the differential nonlinearity of the TDC, producing a systematic error between even and odd codes [13]. This effect has been included in the model by applying a non-uniform rounding to the generated timestamps before being transformed into TDC codes. By exploiting the temporal correlation between photons it is possible to reduce the intensity of the background noise and recover the signal peak. Figure 15 shows the resulting histogram after the temporal correlation of $N_{ph}=2$ photons when a time window $T_{win}≃$ 2.3 ns is applied. As it can be noticed, the residual noise level is slightly higher in the measured data histogram, while the signal peak intensity is well reproduced. This effect can be explained considering the joint effect of crosstalk between the eight SPADs inside each pixel and afterpulsing due to the short dead-time. Those non-idealities have almost no effect when no temporal correlation between photons is considered, since the ToF timestamp is given by the first detected event. On the other hand, when the timestamp is extracted after the temporal correlation of detected photons, the additional events due to crosstalk and afterpulsing will increase the probability to have two or more events occurring inside the correlation time window that will be considered to be valid for the detection process.

## 6. Conclusions

A comprehensive, complete, Montecarlo simulator for direct-ToF systems has been presented and validated against an existing setup. All of the components of a complete ToF detection system are described and modeled in order to provide a solution to foresee the final product in terms of both costs and achievable performance. Many different pixel structures and detection paradigms can be easily included in the simulation flow allowing the possibility to implement the most promising solution already in the design phase of the detector. The capability of the simulator has been proven against an existing setup, with good match between simulated and measured data. Future improvements are aimed to both improve the completeness of the already existing model and include new features. In particular, we aim at improving the SPAD detection model by including non-idealities, such as crosstalk and afterpulsing effects. From a statistical point of view, crosstalk and afterpulsing effects create correlated events that are non-trivial to be efficiently modeled. Regarding afterpulsing in particular, probabilistic models and characterization techniques which may be employed in the simulation process have been proposed [35,36], even though the universality of other approaches is questioned in the work from Ziarkash et al [37]. Therefore, our strategy will be toward the investigation of the most suited approach which provides statistically reliable results with a computationally efficient implementation. Eventually, we foresee extending the simulation environment to include different types of detectors other than SPADs, in particular SiPM and APDs, to broaden the range of application of the simulator.

## Figures and Tables

**Table 1:** Simulation parameters.

Parameter	Value	Unit
**Pixel**		
PDP ${}^{†}$	25	%
Fill factor	26.5	%
Pixel Area	3600	μm^2^
Dead time	15	ns
Median DCR ${}^{‡}$	6.8	kHz
TDC LSB	250	ps
**Emitter**		
Central wavelength	405	nm
Pulse energy	6.2	pJ
Pulse FWHM	≃250	ps
Beam divergence	≃1.7	${}^{\circ }$
**Optical elements**		
Filter bandwidth FWHM	10	nm
Transmittance	66	%
Focal length	6	mm
Diameter	5	mm
**Environment**		
Reflectivity	75	%
Background flux	≃90	MPh/s/pixel
**System**		
Nr. histogram points	250	
Global jitter FWHM ${}^{†}$${}^{†}$	1500	ps

${}^{†}$ Value from [33]. ${}^{‡}$ Value from [13]. ${}^{†}$${}^{†}$ estimated from the entire measurement setup.
